# Neural activity during solo and choral reading: A functional magnetic resonance imaging study of overt continuous speech production in adults who stutter

**DOI:** 10.3389/fnhum.2022.894676

**Published:** 2022-07-22

**Authors:** Emily O. Garnett, Ho Ming Chow, Sarah Limb, Yanni Liu, Soo-Eun Chang

**Affiliations:** ^1^Michigan Medicine, Department of Psychiatry, University of Michigan, Ann Arbor, MI, United States; ^2^Department of Communication Sciences and Disorders, University of Delaware, Newark, DE, United States

**Keywords:** stuttering, fMRI, functional connectivity, speech fluency, continuous speech, default mode network, auditory motor integration

## Abstract

Previous neuroimaging investigations of overt speech production in adults who stutter (AWS) found increased motor and decreased auditory activity compared to controls. Activity in the auditory cortex is heightened, however, under fluency-inducing conditions in which AWS temporarily become fluent while synchronizing their speech with an external rhythm, such as a metronome or another speaker. These findings suggest that stuttering is associated with disrupted auditory motor integration. Technical challenges in acquiring neuroimaging data during continuous overt speech production have limited experimental paradigms to short or covert speech tasks. Such paradigms are not ideal, as stuttering primarily occurs during longer speaking tasks. To address this gap, we used a validated spatial ICA technique designed to address speech movement artifacts during functional magnetic resonance imaging (fMRI) scanning. We compared brain activity and functional connectivity of the left auditory cortex during continuous speech production in two conditions: solo (stutter-prone) and choral (fluency-inducing) reading tasks. Overall, brain activity differences in AWS relative to controls in the two conditions were similar, showing expected patterns of hyperactivity in premotor/motor regions but underactivity in auditory regions. Functional connectivity of the left auditory cortex (STG) showed that within the AWS group there was increased correlated activity with the right insula and inferior frontal area during choral speech. The AWS also exhibited heightened connectivity between left STG and key regions of the default mode network (DMN) during solo speech. These findings indicate possible interference by the DMN during natural, stuttering-prone speech in AWS, and that enhanced coordination between auditory and motor regions may support fluent speech.

## Introduction

Robust connectivity and interactions among cortical auditory and speech-motor brain areas provide the basis for speech production. Auditory-motor integration is crucial for fluent speech, which is disrupted in disorders such as developmental stuttering. Stuttering affects 1% of the population and manifests as frequent involuntary interruptions in the speech flow such as repetitions (i.e., sound/syllable repetitions) and dysrhythmic phonations (i.e., blocks and prolongation of sound/syllables). Decades of behavioral and neuroimaging research have offered accounts of inefficient or disrupted auditory motor integration in adults who stutter (AWS). Key findings come from several lines of research that center on the application or modulation of sensory input during speech production, including reduced motor adaptation in response to auditory perturbations (Cai et al., 2012, 2014; Daliri et al., 2018; Daliri and Max, 2018) and near-elimination of speech disfluencies under rhythmic pacing or delayed auditory feedback conditions (Barber, 1940; Azrin et al., 1968; Hutchinson and Norris, 1977; Stager et al., 1997; Toyomura et al., 2011). Further evidence for disrupted auditory motor integration comes from studies using neuroimaging methods such as functional magnetic resonance imaging (fMRI) that show aberrant brain activity and/or connectivity among speech and auditory brain areas (for review see Chang et al., 2019). In particular, findings from the neuroimaging literature include *overactivation* of cortical speech motor regions, particularly in the right hemisphere, but *decreased* activation in auditory regions, during speech production, both of which become attenuated under fluency induced conditions or following intensive fluency training (Braun, 1997; Stager et al., 2003; Brown et al., 2005; Toyomura et al., 2011, 2015; Budde et al., 2014). Together, these findings suggest that disruption in auditory-motor integration may have a negative impact on generating fluent speech.

The auditory system plays a crucial role in speech production (Guenther and Hickok, 2015) reflected in traditional and as well as newer neurocomputational models. For example, according to the dual route speech processing model (Hickok, 2012), the ventral and dorsal streams work in parallel to integrate sound into both meaning and action, respectively, utilizing the very nature of a speech target, the auditory signal, as a corrective speech output tool. The Directions into Velocities of Articulators [(DIVA); (Bohland and Guenther, 2006; Tourville and Guenther, 2011] model consists of a feedforward control system, which generates already established motor commands guiding speech production, and a feedback control system that provides online detection of production errors by comparing the incoming auditory signal to the expected auditory signal. These auditory targets are represented in the auditory state map in posterior auditory cortex. The superior temporal gyrus (STG) is part of this auditory feedback control system that through an inverse mapping process transforms the auditory target to motor commands in the motor areas using robust structural connections to the ventral motor cortex *via* the feedback control map in right ventral premotor cortex. Importantly, as these systems develop and become established, speech production is achieved primarily through a strong feedforward system, with less of a role of the auditory cortex and feedback control (Bohland and Guenther, 2006; Tourville and Guenther, 2011; Kearney and Guenther, 2019).

Indeed, several theoretical perspectives on stuttering have hypothesized that stuttering is due to an over-reliance on auditory feedback (Max et al., 2004; Civier et al., 2010, 2013), particularly as a result of impaired feedforward control mechanisms, although others propose that the issue arises in the feedback control system itself (e.g., (Max and Daliri, 2019). Recently, using the GODIVA model, Chang and Guenther (2020) proposed that the key impairment underlying stuttering is in the feedforward system, specifically in the cortico-basal ganglia loop associated with initiating speech motor programs, similarly proposed also by others in the field (Maguire et al., 2002, 2004; Alm, 2004; Chang and Zhu, 2013; Civier et al., 2013). Importantly, the auditory system can influence motor behavior specifically through these *corticostriatal* projections (Znamenskiy and Zador, 2013). As noted in Chang and Guenther (2020), auditory feedback of self-generated speech may not match the target auditory pattern for a speech sound due, for example, to minor articulation errors. In this case, this mismatch between expected and actual sensorimotor context may impair crucial initiation commands by the basal ganglia, leading to stuttering. In this context, inhibiting auditory feedback of one’s own speech to avoid detection of minor errors during production may help reduce the mismatch and allow the basal ganglia to generate initiation signal to allow fluent speech. Such an account is consistent with one of the most commonly reported neuroimaging findings in AWS, namely decreased activation in auditory regions during speech tasks. Conversely, auditory activity in AWS becomes comparable or even exceeds levels observed in non-stuttering adults under (or after) fluency inducing conditions such as choral speech or fluency shaping (Fox et al., 1996; Ingham, 2003; Stager et al., 2003; De Nil et al., 2008; Toyomura et al., 2011). Such observations suggest that studies delving further into the mechanisms by which fluency-inducing conditions modulate brain activity in speech-motor and auditory regions may lead not only to a better general understanding of the biological basis of stuttering but may also inform current treatment strategies.

Fluency inducing conditions include speaking in unison with another person, metronome-timed speech, singing, masking, or listening to transformed sensory (auditory) feedback of one’s own voice (Andrews et al., 1982; Bloodstein and Ratner, 2008; Frankford et al., 2021). Such techniques have several factors in common. First, the effects are robust but temporary (Kalinowski and Saltuklaroglu, 2003). Second, they typically involve an external pacing component. In choral speech, this is represented by the other speaker’s reading pattern and pace. The person who stutters then speaks in unison with the other speaker, which drastically reduces their stuttering. In paced speech the external component is represented by a metronome, for example, and the person who stutters matches the timing of their own speech (typically at the syllable or word level) to the beat of the metronome, again resulting in perceptually fluent speech. One proposed account for this “rhythm effect” (Barber, 1940; Azrin et al., 1968; Stager et al., 1997; Toyomura et al., 2011, 2015; Davidow, 2014; Frankford et al., 2021) is that stuttering stems from an inefficient or disrupted *internal* timing mechanism, whereby the addition of an external rhythm allows speech production to bypass the faulty internal mechanism and proceed using the external pace (Alm, 2004; Etchell et al., 2014). Under these external pacing conditions speech production proceeds fluently, resulting from better matching between expected and actual incoming sensory input. With the improved speech timing, the feedforward control mechanism can guide speech production, rather than over-relying on the feedback control system (Civier et al., 2013).

Third, fluency inducing conditions seem to reduce brain activity differences observed during stutter-prone speech. Namely, speaking under conditions that involve external pacing results in increased left frontotemporal activation, and reduced motor hyperactivity, including in the right frontal opercular areas (De Nil et al., 2003; Neumann et al., 2005; Giraud, 2008; Kell et al., 2009; Toyomura et al., 2011, 2015). In particular, STG consistently shows increased activity under fluency inducing conditions suggesting that this region plays an integral role in facilitating fluent speech in people who stutter.

One critical limitation of the aforementioned research findings is that the studies primarily used covert speech, single words, and/or short phrases as speech production tasks while capturing functional brain activity. The use of truncated speech, often incorporating sparse scanning paradigms, were required due to the motion artifacts associated with continuous speech that severely affects the fMRI signal. These paradigms are limited because stuttering typically does not occur on single words; sentence-level or longer utterances are needed to capture brain activity patterns that differentiate stutterers from non-stutterers. Moreover, single word tasks may not fully elucidate brain areas involved in fluency inducing conditions.

To address this gap in the field, we used a validated fMRI artifact removal technique designed specifically for continuous speech production studies to explore brain activity in AWS during continuous natural speech and under fluency inducing conditions. This technique effectively removes speech-related movement artifacts in fMRI data, allowing us to capture brain activity patterns during overt, continuous speech production (AbdulSabur et al., 2014; Xu et al., 2014). In this study, we examined brain activity during choral reading (fluency-inducing) and solo reading (prone to disfluencies) in AWS. Among the potential fluency-inducing conditions, we chose to use choral speech for the following reasons. First, past neuroimaging investigations examining brain activity differences during fluent and induced fluent speech had primarily involved reading and choral speech conditions (Fox et al., 1996, Braun et al., 1997; Fox et al., 2000; Ingham et al., 2012). One reason for this is that metronome or other similarly paced conditions could lead to unnatural sounding speech. Second, we were concerned about the possible interaction between the regular pulse sounds of the scanner and the rhythmic sounds of the metronome. Third, designing a condition that controlled for the auditory feedback of the metronome to be applied during the solo condition was also challenging. Finally, our primary aim was to examine how brain activity patterns and functional connectivity of the auditory cortex differ between an induced fluency condition that involves external rhythmic stimuli (choral reading) and a condition that relies on the speaker’s internal timing ability (solo reading).

Guided by previous findings, we hypothesized that relative to controls, fluency-induced speech in AWS would be associated with increased activity in the auditory regions including posterior STG, and reduced hyperactivity in motor cortical regions including the IFG, premotor, and motor cortical areas. We further hypothesized that compared to natural speech, fluency-induced speech would be associated with greater functional connectivity between left STG and speech motor areas in AWS. Although we primarily focused on the neurophysiological effects of choral reading, we also examined the behavioral effects, namely the effectiveness of choral reading in reducing stuttering. We therefore expected that choral reading would lead to a greater reduction in amount of stuttering compared to solo reading; however, it is likely that stuttering will occur only rarely in either reading condition due to the masking effects of the scanner noise. Such effects, however, are constant across the solo and choral conditions, and would not preclude investigation of the primary research question, which was to examine the effects of rhythmic pacing that would be provided by the choral and not the solo condition.

## Materials and methods

### Participants

Thirty-one adults participated in this study, 15 AWS (4F) and 16 adults (4F) who did not stutter (controls). Detailed demographic information can be found in Supplementary Table 1. All participants were native English speakers who reported no speech, language, hearing, cognitive, or psychiatric disorders, other than stuttering for the AWS group. Groups did not differ significantly in age or expressive or receptive language. The AWS group reported slightly higher years of education (*M* = 14.8) than the control group (*M* = 13.43; *p* = 0.04). Stuttering severity was obtained by certified speech-language pathologists (SLPs) using the Stuttering Severity Instrument (SSI-4; Riley, 2009), and ranged from very mild to very severe based on SSI-4 composite scores. The protocol was approved by the Institutional Review Boards of the University of Michigan Medical School. All subjects gave written informed consent in accordance with the Declaration of Helsinki.

### Study design

Participants were recruited as part of a larger within-subject double-blind study that investigated the effects of transcranial direct current stimulation (tDCS) paired with speech fluency training on brain activity (Garnett et al., 2019). In the present study, only the fMRI scans acquired before stimulation were analyzed, to eliminate possible influences of tDCS or speech fluency training.

#### Stimuli and task

Stimuli were recordings of short paragraphs spoken by a native English male speaker in a neutral tone at approximately 155 words per minute (Chow et al., 2014, 2015). The passages were assessed to be at a 7th grade Flesch-Kincaid Grade Level. The current study used 16 unique paragraphs, each 30 s in length. Additionally, a second recording was created in which each paragraph was played backwards to be used during solo reading (see below). Therefore, there were 32 paragraphs in total (16 forward, 16 backward). These were divided into 4 unique sets to correspond to the 4 runs of the fMRI experiment. Each run began with a 14-s fixation cross, followed by eight 30-s trials, one paragraph per trial. Within each run, participants read 4 paragraphs under “solo reading” and the same 4 paragraphs under “choral reading” conditions, in an alternating fashion. During choral reading, participants read the paragraph shown on the screen while matching their reading pace with an audio recording of the same passage presented *via* MRI-compatible earphones. During solo reading, participants read the passage naturally while a recording of the passage was played backwards via the earphones. The reversed speech recording was used to match the level of external auditory feedback delivered between the solo and chorus conditions, while not inducing speech pacing for the solo condition.

Each trial consisted of a brief instruction screen lasting 3 s that indicated if the subject should “read alone” (solo reading) or “read together” (choral reading) when the next paragraph appeared on the screen. Following the instructions, there was a 3 s fixation cross, after which the paragraph appeared on the screen for 30 s. There was 16 s of fixation cross at the end of the run. Each run lasted approximately 7 min. See Supplementary Figure 1 for an example trial. Prior to the fMRI session, participants completed practice trials with corrective feedback. Participants wore over-the-ear headphones as well as ear plugs during scanning. Additionally, a noise canceling microphone was placed close to the mouth to capture participants’ speech, and a flexible camera was placed over the participants’ mouth to separately capture video during speech.

### Functional magnetic resonance imaging parameters, processing, and analysis

#### Functional magnetic resonance imaging acquisition

The fMRI data were acquired using a 3T GE MRI scanner (MR 750). A standard echoplanar (EPI) pulse sequence was used, with the following parameters: repetition time (TR) = 2 s; echo time (TE) = 30 ms, flip angle = 90°, in-plane resolution = 3.4 × 3.4 mm; 37 interleaved sagittal slices; slice thickness = 4 mm, acceleration factor = 2. In addition, high-resolution structural images were acquired at the beginning of each scanning session using spoiled gradient-recalled acquisition in steady state (SPGR) imaging (TR = 12.236 ms, TE = 5.184 ms, flip angle = 15°, resolution = 1 × 1 × 1mm).

#### Denoising speech-related movement artifacts

SPM12^1^ was used for fMRI data preprocessing and statistical analysis unless specified otherwise. For each participant, functional images were corrected for differences in slice acquisition timings. Anatomical scans and functional volumes were co-registered to the first volume of the first scan using rigid body rotation. Functional scans were concatenated and de-noised using a strategy detailed in our previous publication (Xu et al., 2014). This fMRI denoising technique uses spatial independent component analysis (sICA) to decompose the functional images into a number of independent components and automatically identify and remove noise components based on their spatial patterns (Xu et al., 2014). This technique has been validated using positron emission tomography (PET) and has been demonstrated to be able to remove fMRI artifacts associated with continuous speech production (AbdulSabur et al., 2014; Xu et al., 2014). Because PET is less susceptible to motion artifacts, it is considered the “gold standard” for studying the neural processing of speech production. Xu et al. (2014) study showed that sICA denoising method can effectively remove artifacts associated with speech production and that the results of de-noised fMRI and PET were comparable. De-noised functional scans were normalized to MNI space using DARTEL and spatially smoothed with a 6 mm FWHM kernel (Ashburner, 2007).

#### Task-based functional magnetic resonance imaging

We performed two separate task-based fMRI analyses. First, we compared brain activity *between groups* in each reading condition by examining the contrast *AWS - Control* in the solo reading condition (Section “Brain activity in adults who stutter compared to controls during solo reading”) and the same contrast separately in the choral reading condition (Section “Brain activity in adults who stutter compared to controls during choral reading”). This contrast provides information about potential between-group differences in each of the two reading conditions. Here we expected to observe significant differences between AWS and controls during solo reading, but more similar patterns of activity during choral reading.

Second, we compared brain activity between conditions *within each group* by examining the contrast *Choral – Solo* within the AWS group (Section “Brain activity during choral compared to solo reading in adults who stutter”) and separately within the control group (Section “Brain activity during choral compared to solo reading in controls”). This contrast provides information about how brain activity differs between choral and solo reading in each participant group. For example, one might expect minimal or no differences between choral and solo reading in the control group because there will be no induced fluency effect in this group (as they do not stutter). Conversely, one might expect to see more differences between choral and solo reading in the AWS group, however, given the strong fluency-inducing effect of choral reading on stuttering frequency.

Each participant’s preprocessed data was analyzed using a general linear model (GLM) implemented in SPM12. Reading conditions (choral and solo) were modeled with separate regressors. Individual beta estimates were entered into group level analysis. Statistical threshold was set at voxel-wise *p* = 0.001 and cluster size of 19, corresponding to *p* < 0.05 corrected, using AFNI 3dClustSim (version 17.2.13) with non-Gaussian auto-correlation function (-acf option) (Cox et al., 2017).

#### Task-based functional connectivity

Previous research has reported induced fluency (rhythmic) conditions are associated with heightened STG activity particularly in the left hemisphere in AWS (Salmelin et al., 1998; Stager et al., 2003; Toyomura et al., 2011). Using a seed-based functional connectivity analysis, we asked whether induced fluency during choral reading (relative to solo reading) was associated with increased functional connectivity between left STG and speech motor areas. For this analysis, we selected a left STG seed region with peak coordinates of –50, –34, 18 as reported in Toyomura et al. (2011). In that study, left STG was identified as the key region that showed increased activity in AWS during fluency induced conditions (exceeding activity levels seen in controls) but significantly reduced activity during solo reading. In this analysis we examined areas across the whole brain that showed significantly different functional connectivity the left STG seed region (Section “Functional connectivity”).

For each subject, functional images corresponding to each reading condition were separated and concatenated. Time-series for each condition were band-passed filtered with cutoff frequency at 0.03 to 0.2 Hz. Time-series of the seed region was extracted by averaging voxels in a sphere of 5 mm radius at the coordinates. Pearson’s correlation coefficients were calculated between the time-series of the seed region and the time series of each voxel in the whole brain and converted to Fisher’s z-scores. The individual maps were analyzed using GLM. Using AFNI 3dClustSim, a voxel wise height threshold of *p* = 0.01 and a cluster size of 70 was considered significant, corresponding to a corrected *p* < 0.05.

### Speech rate, loudness, and stuttering frequency during scanning

Speech rate in syllables per second (SPS) was calculated by dividing the number of syllables by total speaking time. Speech rate and %SS were calculated separately for solo and choral reading passages. To assess any differences in loudness between choral and solo reading, a trained study team member blinded to condition and study objectives listened to each passage and rated it from 1 (quietest) to 5 (loudest) separately for each subject. That is, loudness ratings were completed within each subject rather than across subjects, as speaking volume naturally varied across participants. The study team member was blind as to the type of reading passage (solo or choral). This analysis was completed for AWS and control groups.

This analysis was completed for both AWS and control groups. A certified SLP with expertise in stuttering and disfluency analysis listened to the recordings of each passage for each AWS participant to determine stuttering frequency. The SLP was blinded to the condition (choral or solo reading). The total number of syllables was noted and marked for the presence or absence of stuttering, defined as dysrhythmic phonations (prolongations, blocks) and whole word or part-word repetitions. Percent stuttered syllables (%SS) was calculated for the AWS group only, as no participants in the control group stuttered.

## Results

### Speech rate, loudness, and stuttering frequency

Supplementary Table 2 shows between group differences in speech rate and loudness during solo and choral reading. Loudness ratings for two controls and two AWS, speech rate for one control and one AWS, and disfluency rates for one AWS were unable to be calculated due to poor audio recording quality. There were no significant differences between the AWS and control groups in syllables per second (SPS) or loudness in either reading condition (all *p* values > 0.078; see Supplementary Material for details).

Within-group comparisons showed no significant differences in speech rate in solo (*M* = 3.60) compared to choral reading (*M* = 3.64) in the AWS group (*p* = 0.777). The control group on average spoke significantly faster during solo reading (*M* = 3.85) than choral reading (*M* = 3.68, *p* < 0.001; Supplementary Table 3). Both groups spoke slightly but significantly louder in solo reading compared to choral reading (Supplementary Table 3).

In the AWS group, stuttering frequency as measured by percent stuttered syllables (%SS) was comparable in the choral (*M* = 1.36%) and solo (*M* = 1.8%) reading conditions (*p* = 0.777; Supplementary Table 4). However, closer inspection of the individual subjects showed that the subject with the highest SSI score showed a dramatic decrease in %SS during choral reading but maintained a high rate of stuttering during the solo condition. Consequently, we repeated this analysis after excluding this subject. Results showed that %SS was significantly greater in the choral reading condition (*M* = 1.42%) than the solo reading condition (*M* = 0.42%; *p* < 0.001). Given that 3% is an often-used threshold to determine stuttering status, the %SS in both conditions was well below this number. The effect of the scanner noise during speech is likely to have had a strong influence on induced fluency. Therefore, the %SS difference between the two conditions is not considered to be meaningful.

### Task-based activation

#### Brain activity in adults who stutter compared to controls during solo reading

**FIGURE 1:**
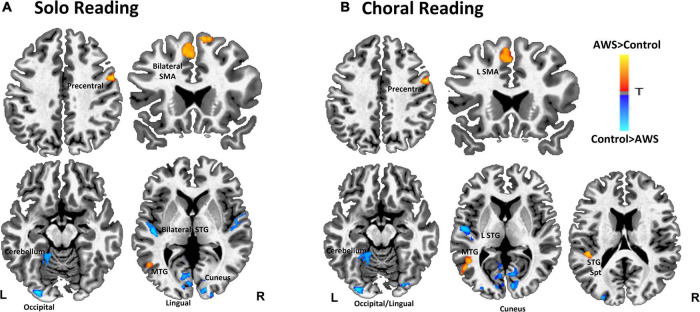
Contrast between adults who stutter (AWS) and control groups during solo reading **(A)** and choral reading **(B)**. Warmer colors represent with significantly greater activity for AWS compared to controls. Statistical map has a threshold at *p* < 0.05 (corrected).

We first compared brain activity between groups during solo reading (Table 1 and Figure 1A). Compared to controls, AWS exhibited heightened activity in right precentral gyrus, bilateral supplementary motor area (SMA), and left middle temporal gyrus (MTG). In contrast, AWS exhibited decreased activity in left cerebellum, occipital/lingual gyri, right cuneus, and bilateral STG.

**TABLE 1:** Group differences in solo reading (left panel) and choral reading (right panel).

Solo reading						Choral reading					
Region	x	y	z	*t*	Voxels	Region	x	y	z	*t*	Voxels
***AWS* > *Controls***						***AWS* > *Controls***					
Precentral (R)	54	3	36	5.02	37	Precentral (R)	54	3	36	5.59	42
SMA (L)	–3	18	48	4.89	31	MTG (L)	–51	–63	3	5.66	35
SMA (R)	9	18	63	4.77	24	SMA (L)	–3	18	48	4.68	30
MTG (L)	–51	–63	3	5.02	21	STG/INS (L)	–45	–36	15	5.37	21
***AWS* < *Controls***						***AWS* < *Controls***					
Lingual (L)	–27	–93	–18	–6.75	119	Lingual (L)	–27	–93	–18	–7.36	192
Cuneus (R)	15	–93	0	–5.52	50	Cuneus (R)	15	–93	0	–5.72	48
STG/HG (L)	–45	–21	3	–6.02	36	STG/HG (L)	–45	–21	3	–5.76	31
Cerebellar Lobules I-IV (L)	–6	–51	–6	–4.9	34	MOC (L)	–24	–93	12	–4.61	24
MOC (L)	–24	–93	12	–4.75	34	Cerebellum (Crus I) (R)	27	–87	–18	–4.97	23
STG/HG (R)	51	–15	3	–4.38	25	Posterior cingulate/cuneus (R)	9	–72	6	–4.26	19

#### Brain activity in adults who stutter compared to controls during choral reading

During choral reading AWS exhibited heightened activity compared to controls in right precentral gyrus, as well as left middle temporal, SMA, and STG/Insular gyri (Table 1 and Figure 1B). Decreased activity for AWS relative to controls was found in left hemisphere lingual, middle occipital, and STG, as well as right cuneus, cerebellum, and posterior cingulate.

#### Brain activity during choral compared to solo reading in adults who stutter

The AWS group exhibited heightened activity for choral compared to solo reading in left angular gyrus, middle frontal gyrus (MFG), right STG/SMG in the area of SPT, right middle cingulate, and bilateral superior frontal gyrus (SFG). Decreased activity during choral relative to solo reading was found in right precuneus, cingulate gyrus, MFG/SFG, and insula, left IFG, and the cerebellar declive. See Supplementary Table 5 (top panel) for details of cluster sizes, coordinates, and test statistics, and Supplementary Figure 2A for activity patterns.

#### Brain activity during choral compared to solo reading in controls

Controls exhibited heightened activity during choral relative to solo reading in left anterior cingulate and cerebellar crus I, right middle cingulate (extends into L), and bilateral angular gyrus and MFG. Controls exhibited decreased activity during choral relative to solo reading in left cerebellar crus II, STG, precuneus, and insula extending into the caudate, right SFG/MFG, IFG/insula, SMA, and MFG, and bilateral superior parietal lobe. See Supplementary Table 5 (bottom panel) for details of cluster sizes, coordinates, and test statistics, and Supplementary Figure 2B for activity patterns.

### Functional connectivity

**FIGURE 2:**
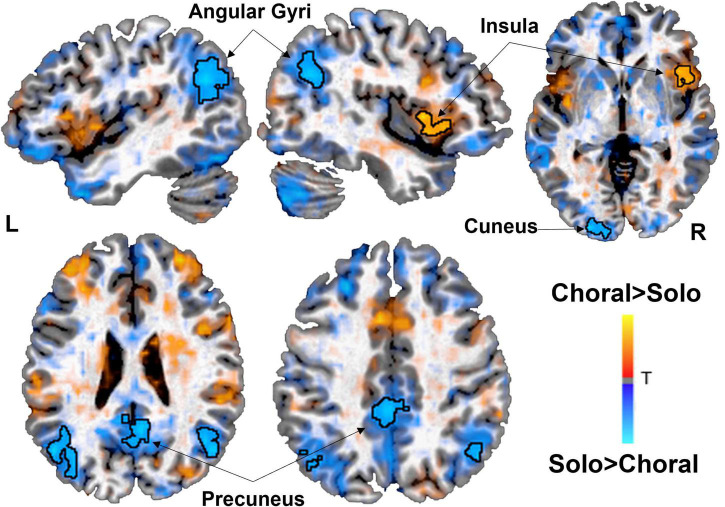
Brain areas showing significant functional connectivity with the left superior temporal gyrus (STG) seed during choral (warm) vs. solo (cold) reading in adults who stutter (AWS). During choral (fluency induced) reading, the left auditory region showed increased correlated activity patterns with the right insula/inferior frontal gyrus. During solo (stuttering prone) reading, the left auditory region increased functional connectivity with the bilateral angular gyri, cuneus, and precuneus, areas that are part of the default mode network. There were no significant functional connectivity findings in the control group. Statistical map has a threshold at *p* < 0.05 (corrected).

Functional connectivity analyses were conducted within each group for choral versus solo reading. Results showed that AWS exhibited increased connectivity between left STG and right insula in the IFG (and left IFG detected sub-threshold; Figure 2) during choral versus solo reading condition. On the other hand, functional connectivity of left STG was significantly increased in the bilateral angular gyri and precuneus for AWS during the solo condition relative to the choral reading condition (Table 2 and Figure 2). In the control group, functional connectivity of the left STG did not differ significantly between the two speech conditions (not shown).

**TABLE 2:** Regions showing significant functional connectivity in AWS using an a *priori* determined left superior temporal gyrus (LSTG) seed (Toyomura et al., 2011).

Region	x	y	z	*t*	Voxels
***Choral* > *Solo***					
Insula (R)	42	6	3	4.7	78
***Solo* > *Choral***					
Precuneus	6	–54	18	–4.3	197
Angular Gyrus (L)	–48	–68	27	–4.4	187
Angular Gyrus (R)	51	–66	24	–4.2	108
Cuneus/middle occ. (L)	–12	–99	0	–4.3	72

## Discussion

A major aim of this study was to investigate how brain activity patterns in the auditory cortex during continuous speech production differ between AWS and controls. Overall, group differences in brain activity patterns observed in each condition were largely similar, showing the expected pattern in AWS of heightened activity in motor areas (right hemisphere premotor cortex and SMA) but decreased activity in auditory regions previously reported as neural signatures associated with stuttering (Brown et al., 2005; Belyk et al., 2015; Neef et al., 2015). In this way, the current results partially support our hypothesis that choral reading would attenuate the aberrant motor and auditory activity during speech in AWS relative to controls; however, these activity pattern differences were subtle. For example, compared to controls, AWS exhibited heightened bilateral SMA during solo reading, but only left SMA showed this pattern during choral reading. Additionally, solo reading was associated with decreased activity in bilateral STG, yet during choral reading only left STG showed decreased auditory activity in AWS relative to controls.

When directly comparing choral and solo reading in AWS and controls separately, the induced fluency condition in AWS was associated with a pattern of greater brain activity in areas in bilateral STG/SMG, angular gyrus, and MFG, regions linked to the executive control network. The solo condition was associated with greater brain activity in areas linked to the cingulo-opercular network in AWS, which supports maintaining sustained goal oriented cognitive control. In contrast, controls exhibited greater activity in anterior and middle cingulate in the choral relative to the solo condition. Choral reading was also associated with decreased activity in bilateral parietal regions as well as several important motor speech regions including SMA and the IFG/insular area.

An interesting convergent finding in the task-based fMRI activity was that induced fluency seemed to be associated with increased activity in a cluster in posterior STG bordering the temporal parietal junction showed increased activity for AWS relative to controls. This cluster (using both peak [–45, –36, 15] and center of mass [–43.0, –37.3, 15.1] coordinates) falls within the range of coordinates for area Spt (Buchsbaum et al., 2001, 2005; Hickok et al., 2003, 2009, 2011) arguably a crucial region for auditory motor integration. In their SFC model, Hickok et al. (2011) refer to stuttering as reflecting “noisy” mapping between auditory and motor systems resulting in inaccurate predictions and, consequently, inaccurate corrective commands [see also (Max et al., 2004)]. Moreover, a significant cluster in the vicinity of Spt in the *right* hemisphere was also significantly increased during choral relative to solo speech in the AWS group. Thus, heightened activity in left Spt in AWS relative to controls during choral reading may indicate improved mapping between auditory and motor systems.

Though the task-based activity contrast analyses did not reveal substantial differences, a clear difference emerged between choral and solo reading based on the functional connectivity analysis. Here we examined brain areas showing significantly correlated activity with that of a left STG region previously linked to heightened activity in AWS during a rhythmic speaking task (Toyomura et al., 2011). A novel finding was that during solo speech in AWS, there was heightened connectivity between left STG and key regions of the default mode network (DMN), including bilateral angular gyri and precuneus. Defined based on its correlated activity at rest, DMN is often associated with mind wandering, prospection, theory of mind, and autobiographical memory (Buckner et al., 2008; Spreng et al., 2009). The DMN shows anti-correlations with task-positive networks such as those supporting attention, executive control, and somatomotor functions (Lee et al., 2012). It has also been suggested that performing fluid, automatic motor tasks characteristic of well-learned and skilled movements can break down when attention is focused inwardly to oneself (linked to DMN) versus outwardly toward a movement target (linked to motor networks) (Wulf and Lewthwaite, 2016). Accordingly, efficiently switching from DMN to sensorimotor networks might be expected to support fluent speech production. The significantly increased functional connectivity between DMN-linked regions and left STG that was only present in AWS during solo reading may indicate possible interference of the DMN during natural, continuous speech in AWS (Sonuga-Barke and Castellanos, 2007).

This notion is supported by our previous work showing that children who stutter exhibit aberrant connectivity between DMN and speech and attention networks, and in particular that anomalous connectivity involving DMN predicted persistent stuttering (Chang et al., 2018). Specifically, in that study, connectivity between the somatomotor network (SMN) and the DMN was one of the inter-network connectivity differences that predicted stuttering status. In particular, STG within the SMN showed heightened connectivity with a number of DMN nodes. The SMN on the other hand also showed aberrant connectivity with the attention networks (dorsal and ventral attention networks). Persistence in stuttering was found to be predicted primarily through intra- and inter-network connectivity involving the DMN and its connections to attention and executive control networks. In the present study, the AWS group is by definition a group of adults with persistent stuttering. Interference from the DMN has also been implicated in other neurodevelopmental disorders besides stuttering. For example, in adults with ADHD, hyperactivity of DMN has been shown regardless of task (Cortese et al., 2012), supporting the default mode interference hypothesis (Sonuga-Barke and Castellanos, 2007). For stuttering, hyperconnectivity between the DMN and SMN may reflect heightened internal focus on one’s speech that leads to de-automatized speech patterns that are prone to breakdown. Well-learned motor tasks are performed optimally when focus is on the movement goal (externally focused attention), rather than when excessive inward attention is paid to one’s articulators (which can lead to movement breakdown, and “choking” as documented in athletes under pressure). Supporting this notion, some past reports have shown that stuttering could be reduced in dual task conditions where working memory and attention were manipulated during speaking tasks (Eichorn et al., 2016, 2019). Such dual tasking effects on speech were present regardless of working memory load, suggesting that a general attention allocation away from speaking might be sufficient to increase fluency in speakers who stutter. This may mean that if stuttering speakers can better disengage their somatomotor networks from DMN, better fluency might be achieved. Because the present study did not systematically examine inter-network connectivity between DMN and task positive networks including SMN, however, these interpretations in the context of the present results are speculative and will need to be confirmed in future studies.

During choral relative to solo speech, AWS exhibited increased functional connectivity between left STG and right insula extending into IFG. This finding is partially in line with a recent study investigating the effects of an intensive fluency shaping treatment program on neurofunctional reorganization (Korzeczek et al., 2021). In that study, the intervention strengthened connectivity involving *a priori* defined hubs with a sensorimotor integration network, in particular between left IFG and right pSTG. Right frontal areas have also been associated with feedback control in the DIVA model: if there is a mismatch between expected and actual sensory feedback, the feedback control map in the right frontal/ventral premotor cortex issues an error signal. During auditory and somatosensory perturbation experiments (Tourville et al., 2008; Golfinopoulos et al., 2011), compensation for the perturbations was associated with an increase in right lateralized frontal activity. Therefore, it is possible that corrective actions to motor plans, which can be found during compensatory movements during perturbation and during induced fluency conditions like choral speech, is reflected by increased communication between temporal and frontal regions. More research is needed to examine specific roles of bilateral IFG and STG in stuttering, their functional connectivity with other regions during normal and induced fluency conditions, and how these change as a result of treatment or natural recovery.

Turning briefly to the speech patterns exhibited by AWS during scanning, the results were not completely in line with our hypotheses. We expected the choral reading condition to significantly decrease stuttering to a greater degree than the solo reading condition, but we found the opposite pattern. Importantly, however, *both reading conditions* showed very little stuttering, less than 2%. One potential explanation is that at times, the process of attempting to speak in unison with the recording during choral reading resulted in speech “adjustments” such as slowing a specific sound in order to stay in pace with the recording. Although we did not calculate %SS for the control group as a whole, we tested this hypothesis by having a study team member blinded to group assignment listen and calculate %SS for three control subjects. A similar phenomenon was observed in these three control subjects, none of whom stuttered. Therefore, we speculate that the apparent higher %SS in the choral reading condition was an artifact of attempts at pacing with the audio recording.

Both AWS and control groups spoke louder during solo compared to choral reading. This finding is potentially consistent with the Lombard effect, an innate tendency to speak louder in noisy environments (Lombard, 1911). However, such an account is not so straightforward, given that the overall auditory environment (i.e., scanner noise, bone-conduction, presence and loudness of auditory feedback in the headphones) was comparable in both solo and choral reading conditions. Although we cannot rule out that participants expended greater speech effort during the solo condition in an attempt to hear their own voice more clearly, it does not appear to differ between AWS and controls in this study. It is also possible that the increased loudness during solo reading reflects attempts to “ignore” the reversed speech being played. While this also cannot be ruled out, participants were specifically instructed to speak at approximately the same pace during solo reading as they did during the choral reading condition, so as to remain engaged in overt speech for the same amount of time (i.e., for the 30 s that the text appeared on the screen). In this way, they could not simply tune out the reversed speech or their speech rate would have differed wildly between conditions, as they would likely have reverted to speaking at their natural rate. When examining the speech rate in syllables per second (SPS), while the controls spoke somewhat faster during the solo condition compared to the choral condition, the AWS did not, nor were there significant differences between groups in SPS in either solo or choral reading.

## Limitations

This is the first report comparing brain activity during continuous solo and choral reading in AWS captured with fMRI and using advanced de-noising techniques. Despite some strengths, several important limitations exist. Our sample size was modest and may have contributed to observing overall similar activation between reading conditions, which was seen even at the individual subject level. On the other hand, the differences observed were in line with expectations of reduced motor hyperactivity and increased auditory activity. Because of the novelty of our task and the sICA denoising method, it is difficult to directly compare the current results with those reported in previous studies.

For our functional connectivity analysis, we chose the STG peak showing the greatest change in Toyomura et al. (2011) which was found in the Rhythm vs. Solo contrast. We note their Rhythm condition consisted of metronome-paced speech, which differs from the fluency-inducing condition in the present study. Therefore, it is possible that using different seeds might reveal greater differences in activity patterns between natural and fluent speech, which should be explored in future studies. Nevertheless, our results support the view that increased sensorimotor integration – as evidenced by our induced fluency choral reading condition – is associated with improved neural communication between auditory and motor regions.

## Conclusion

This study leveraged an advanced fMRI de-noising method to allow us to investigate brain activity patterns during continuous speech in adults who stutter and controls under choral and solo reading conditions. Overall, brain activity differences between AWS relative to controls in the two conditions were similar, showing expected patterns of hyperactivity in premotor/motor regions but underactivity in auditory regions. Functional connectivity of left STG showed that within the AWS group there was increased correlated activity with the right insula during choral speech, as well as heightened connectivity with regions of DMN during solo speech. These findings suggest that induced fluency conditions specifically modulated brain activity in the AWS group. Further, they indicate possible interference by the DMN during natural, stuttering-prone speech in AWS, and that enhanced coordination between auditory and motor regions may support fluent speech. These findings have clinical implications for designing interventions that involve fluency-inducing conditions to treat stuttering.

## Data availability statement

The raw data supporting the conclusions of this article will be made available by the authors, without undue reservation.

## Ethics statement

The studies involving human participants were reviewed and approved by the Institutional Review Board of the University of Michigan Medical School (IRBMED). The patients/participants provided their written informed consent to participate in this study.

## Author contributions

EG, S-EC, and HC contributed to the concept and design of the study. EG and HC collected to the data. EG, S-EC, HC, YL, and SL contributed to the analysis and interpretation of results and drafted the manuscript. All authors contributed to the article and approved the submitted version.

## Conflict of interest

The authors declare that the research was conducted in the absence of any commercial or financial relationships that could be construed as a potential conflict of interest.

## Publisher’s note

All claims expressed in this article are solely those of the authors and do not necessarily represent those of their affiliated organizations, or those of the publisher, the editors and the reviewers. Any product that may be evaluated in this article, or claim that may be made by its manufacturer, is not guaranteed or endorsed by the publisher.

## Footnotes

1
https://www.fil.ion.ucl.ac.uk/spm/software/spm12/
