# Supplementary material for: Neural activity during solo and choral reading: A functional magnetic resonance imaging study of overt continuous speech production in adults who stutter
Source: Front Hum Neurosci. 2022 Jul 22;16:894676. doi: 10.3389/fnhum.2022.894676 (PMC9353050; doi:10.3389/fnhum.2022.894676)

Note: Loudness ratings for two controls and two AWS, speech rate for one control and one AWS, and disfluency rates for one AWS were unable to be calculated due to poor data recording quality.

**Supplementary Table S1. Participant demographic information and behavioral test results.**

| **Measure** | **AWS mean (SD; n=15, 4F)** | **Controls mean (SD; n=16, 4F)** | ***t*** | ***p*** |
| --- | --- | --- | --- | --- |
| Age (years) | 22.9 (7.3) | 21.6 (5.6) | -0.56 | 0.58 |
| PPVT-4 | 110.9 (9.4) | 111.7 (11.0) | 0.20 | 0.84 |
| EVT-2 | 119.7 (17.0) | 119.4 (14.3) | -0.05 | 0.96 |
| Education (years) | 14.80 (2.04) | 13.43 (1.45) | -2.17 | 0.04 |
| %SLD | 8.3 (8.1) | n/a | - | - |
| SSI-4 | 17.7 (9.89) | n/a | - | - |
| Handedness | 2 left/13 right | 1 left/15 right | - | - |

SSI-4 = Stuttering Severity Instrument, fourth edition; PPVT = Peabody Picture Vocabulary Test, fourth edition; EVT = Expressive Vocabulary Test, second edition. %SLD = percent stuttering-like disfluencies.

**Supplementary Table S2. Between group differences in speech rate and speaking volume during scanning.**

|  | Stuttering | Controls |  |  |
| --- | --- | --- | --- | --- |
|  | M (SD) | M (SD) | t | p |
| Rate (SPS) Solo Reading | 3.60 (.49) | 3.85 (.12) | 1.83 | 0.078 |
| Rate (SPS) Choral Reading | 3.64 (.08) | 3.68 (.04) | 1.71 | 0.098 |
| Loudness Solo Reading | 3.28 (.34) | 2.94 (.62) | -1.69 | 0.108 |
| Loudness Choral Reading | 2.68 (.26) | 2.74 (.54) | 0.355 | 0.726 |

**Supplementary Table S3. Within group differences in speech rate and speaking volume during scanning.**

|  |  | Rate (SPS) Solo Reading | Rate (SPS) Choral Reading | t | p |
| --- | --- | --- | --- | --- | --- |
| Stuttering | M (SD) | 3.60 (.49) | 3.64 (.08) | -0.289 | 777 |
| Controls | M (SD) | 3.85 (.12) | 3.68 (.04) | 4.95 | <.001 |
|  |  |  |  |  |  |
|  |  | Loudness Solo Reading | Loudness Choral Reading |  |  |
| Stuttering | M (SD) | 3.28 (.34) | 2.68 (.26) | 4.94 | <.001 |
| Controls | M (SD) | 2.94 (.62) | 2.74 (.54) | 3.99 | 0.002 |

**Supplementary Table S4. Disfluencies produced by AWS in solo and choral reading conditions (with and without outlier subject)**

|  |  | %SS Solo Reading | %SS Choral Reading | t | p |
| --- | --- | --- | --- | --- | --- |
| AWS including outlier | M (SD) | 1.79 (5.17) | 1.36 (.68) | .299 | .770 |
| AWS excluding outlier | M (SD) | .42 (.31) | 1.42 (.68) | -5.51 | <.001 |

**Supplementary Table S5. Within group task-based fMRI results.**

| **AWS Group** | | | | | |  |
| --- | --- | --- | --- | --- | --- | --- |
| **Region** | **x** | **y** | **z** | **t** | **Voxels** | |
| ***Choral>Solo*** |  |  |  |  |  | |
| Angular Gyrus (L) | -36 | -72 | 33 | 6.41 | 309 | |
| MFG (L) | -42 | 24 | 42 | 5.88 | 269 | |
| STG/SMG Right (SPT) | 45 | -57 | 24 | 4.91 | 39 | |
| SFG (R) | 18 | 42 | 48 | 4.31 | 28 | |
| R mid cingulate | 3 | -57 | 30 | 4 | 22 | |
| SFG L | -12 | 63 | 12 | 4.35 | 20 | |
| ***Solo> Choral*** |  |  |  |  |  | |
| Precuneus (R) | 15 | -72 | 39 | -5.63 | 144 | |
| Cingulate Gyrus (R, extends to L) | 3 | 24 | 33 | -5.83 | 126 | |
| MFG/SFG (R) | 30 | 60 | 9 | -5.21 | 111 | |
| Insula (R) | 36 | 21 | -3 | -5.33 | 73 | |
| CB Declive (VI) | -6 | -72 | -24 | -4.73 | 20 | |
| L IFG | -45 | 42 | -15 | 3.98 | 19 | |

| **Control Group** | | | | | |
| --- | --- | --- | --- | --- | --- |
| **Region** | **x** | **y** | **z** | **t** | **Voxels** |
| ***Choral >Solo*** |  |  |  |  |  |
| Anterior cingulate (L) | -3 | 45 | -3 | 7.43 | 702 |
| Middle cingulate (R, extends to L) | 3 | -45 | 33 | 6.37 | 334 |
| Middle frontal gyrus (L) | -21 | 30 | 51 | 6.04 | 328 |
| Angular gyrus (L) | -45 | -60 | 36 | 7.17 | 314 |
| Middle frontal gyrus (R) | 42 | 21 | 42 | 5.24 | 132 |
| Angular gyrus (R) | 48 | -60 | 33 | 6.16 | 127 |
| CB Crus I (L) | -42 | -84 | -39 | 4.79 | 72 |
| ***Solo> Choral*** |  |  |  |  |  |
| Superior parietal (R) | 15 | -63 | 54 | 7.51 | 384 |
| Superior parietal (L) | -18 | -72 | 48 | 5.32 | 70 |
| SFG/MFG (R) | 33 | 45 | 33 | 5.62 | 67 |
| CB Crus II (L) | -9 | -75 | -39 | 6.1 | 65 |
| SMA (R) | 6 | 18 | 45 | 5.64 | 53 |
| STG (L) | -33 | -54 | 9 | 4.69 | 43 |
| SMA (R) | 9 | 12 | 63 | 5.64 | 38 |
| IFG/insula (R) | 33 | 30 | -3 | 5.22 | 37 |
| Precuneus (L) | -27 | -75 | 21 | 4.22 | 35 |
| Insula/caudate (L) | -24 | -15 | 21 | 4.49 | 28 |
| MFG (R) | 33 | -3 | 51 | 4.14 | 28 |
| CB I_IV | 0 | -48 | -27 | 4.89 | 24 |
| MFG/IFG (R) | 33 | 15 | 24 | 4.53 | 23 |

**Supplementary Figure S1. Example experimental trial.**


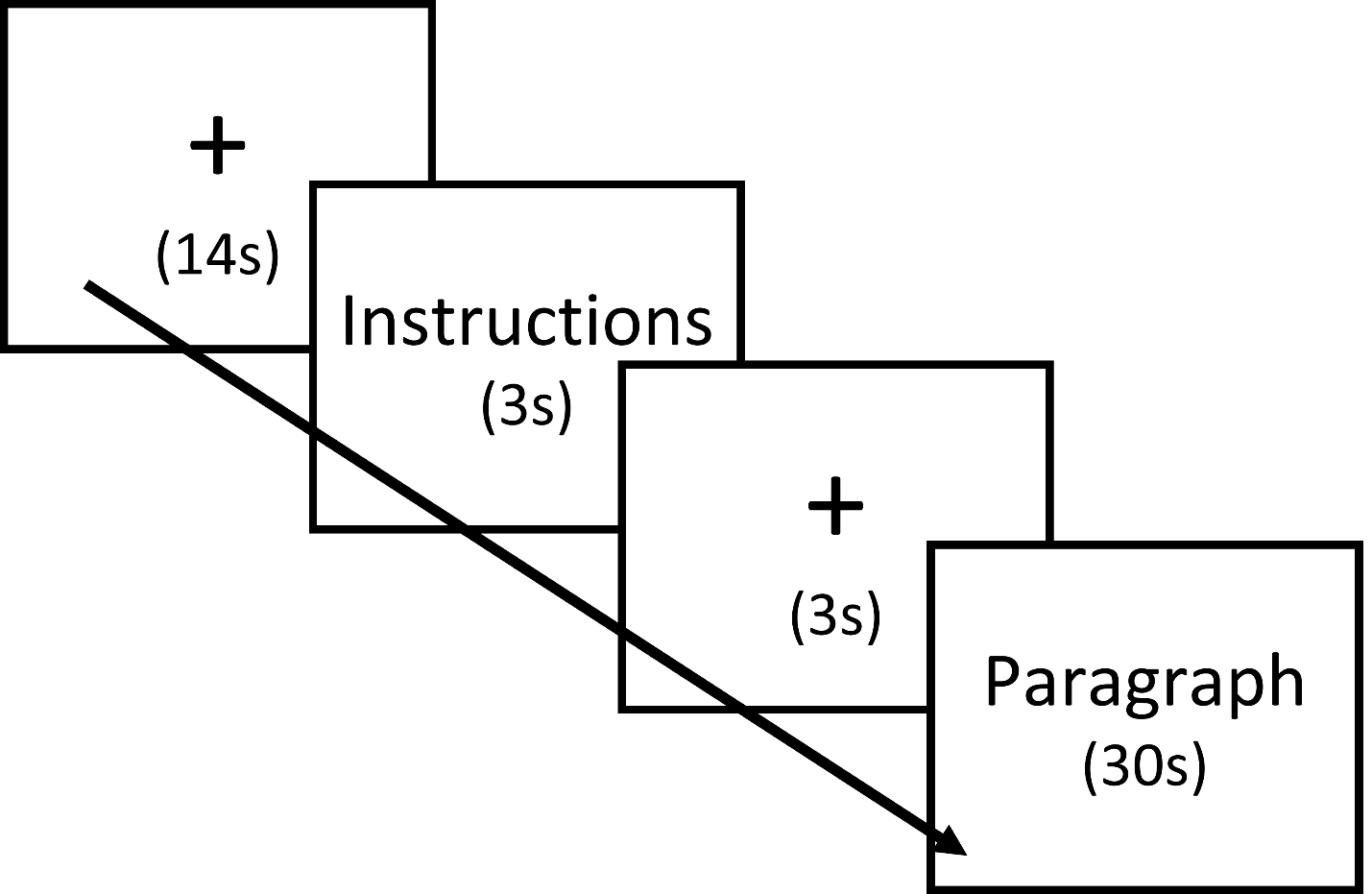


**Supplementary Figure S2. Brain activity during choral compared to solo reading in AWS (A; left) and Controls (B; right).**


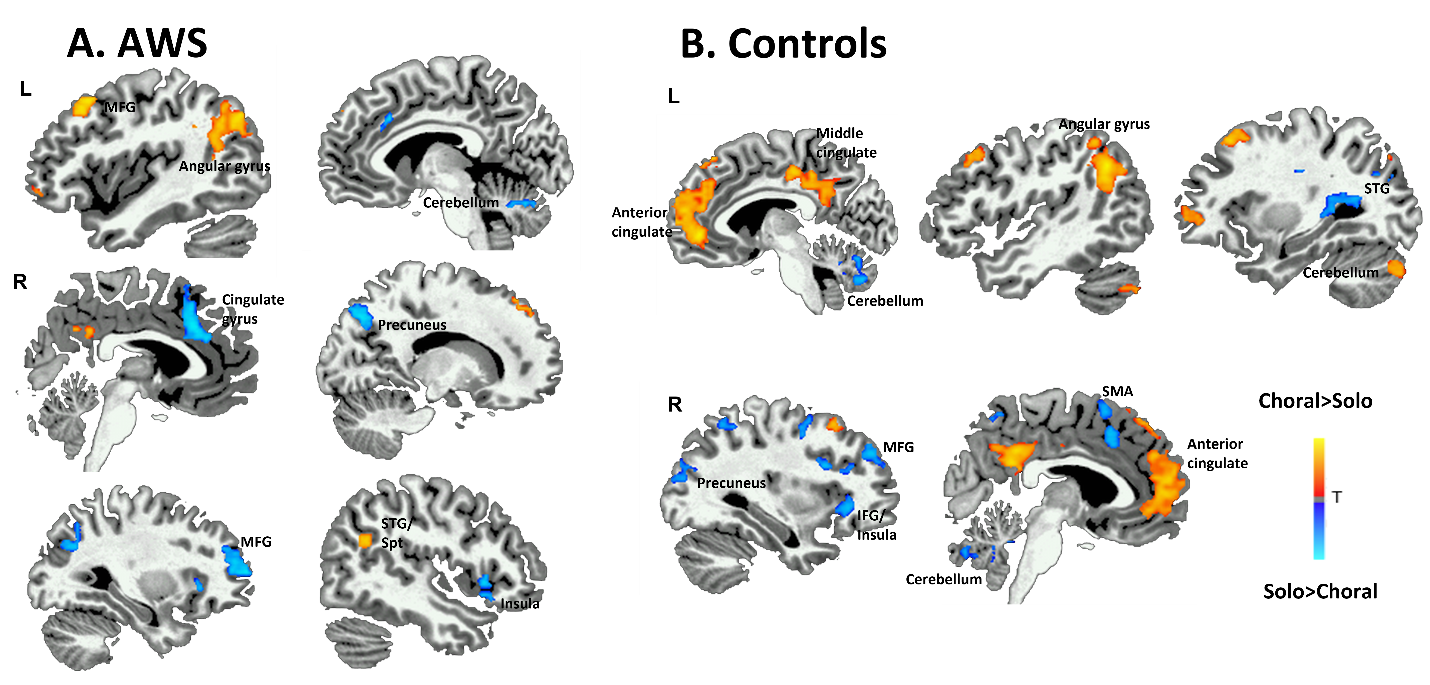

Supplement: Supplementary file 1 [file Data_Sheet_1.docx]
